# A rare case of aortic sinuses of valsalva fistula to multiple cardiac chambers secondary to periannular aortic abscess formation from underlying Brucella endocarditis

**DOI:** 10.3205/dgkh000257

**Published:** 2015-11-10

**Authors:** Feridoun Sabzi, Aghighe Heidari, Reza Faraji

**Affiliations:** 1 Preventive Cardiovascular Research Centre Kermanshah, Kermanshah University of Medical Sciences, Kermanshah, Iran; 2 Department of Anesthesiology, Medical School, Kermanshah University of Medical Sciences, Kermanshah, Iran

**Keywords:** fistula, Brucella, endocarditis, heart surgery

## Abstract

The concomitant presence of abnormal connection from three aortic valsalva sinuses to cardiac chambers is a rare complication of native aortic *Brucella* endocarditis. This case report presents a 37-year-old Iranian female patient who had native aortic valve *Brucella* endocarditis complicated by periannular abscess formation and subsequent perforation to multi-cardiac chambers associated with congestive heart failure and left bundle branch block. Multiple aorto-cavitary fistulas to right atrium, main pulmonary artery, and formation of a pocket over left atrial roof were detected by transthoracic echocardiogram (TTE). She had received a full course of antibiotics therapy in a local hospital and was referred to our center for further surgery. TTE not only detected multiple aorto-cavitary fistulas but also revealed large vegetation in aortic and mitral valve leaflets and also small vegetation in the entrance of fistula to right atrium. However, the tricuspid valve was not involved in infective endocarditis. She underwent open cardiac surgery with double valve replacement with biologic valves and reconstruction of left sinus of valsalva fistula to supra left atrial pocket by pericardial patch repair. The two other fistulas to main pulmonary artery and right atrium were closed via related chambers. The post-operative course was complicated by renal failure and prolonged dependency to ventilator that was managed accordingly with peritoneal dialysis and tracheostomy. The patient was discharged on the 25^th^ day after admission in relatively good condition. The TTE follow-up one year after discharge revealed mild paravalvular leakage in aortic valve position, but the function of mitral valve was normal and no residual fistulas were detected.

## Zusammenfassung

Das gleichzeitige Vorhandensein einer anormalen Verbindung von drei aortalen Valsalva-Nebenhöhlen um die Herzkammern ist eine seltene Komplikation einer aortalen brucellösen Endokarditis. Dieser Fallbericht stellt eine 37-jährige iranische Patientin mit einer nativen brucellösen Endokarditis der Aortenklappe mit Anulusabszess und anschließender Perforation in die Herzkammer vor. Der Fall wurde durch eine kongestive Herzinsuffizienz mit Linksschenkelblock zusätzlich erschwert. Die Patientin wurde nach antibiotischer Vorbehandlung in einem Regionalkrankenhaus an unser Herzzentrum zur weiteren chirurgischen Behandlung überstellt. Mittels transthorakalem Echokardiogramm (TTE) wurden nicht nur mehrere aorto-kavitäre Fisteln, sondern auch weitere große Vegetationen in der Aorta und der Mitralklappe sowie kleine Vegetationen am Eingang der Fistel zum rechten Vorhof nachgewiesen.

Die Patientin erhielt nach Sternotomie in offener Herzchirurgie einen Doppelklappenersatz mit biologischen Klappen, dazu wurde eine linke Sinus valsalva-Fistel durch Perikardpatch saniert. Postoperativ wurde der weitere Verlauf durch ein Nierenversagen und die Notwendigkeit zur mechanischen Beatmung kompliziert. Dennoch konnte die Patientin am 25. Tag nach der Aufnahme in relativ gutem Allgemeinzustand nach Hause entlassen werden.

Die TTE-Nachsorgeuntersuchung 1 Jahr nach Entlassung zeigte eine milde paravalvuläre Leckage in Aortenklappen-Position, während die Funktion der Mitralklappe normalisiert war, und es wurden keine weiteren Restfisteln nachgewiesen.

## Introduction

The etiology of concomitant multiple fistulas originating from three sinuses of valsalva to related cardiac chambers is restricted only to infectious causes. As opposed to multiple aorto-cavitary fistulas, single fistula has more causative factors and includes dissection of ascending aorta, surgery on aortic root, congenital defect between the valsalva sinus and the related chamber, aortic valve replacement, and infective endocarditis [1]. The most common type of aorto-cavitary fistula is the connection of none or right sinus of valsalva to right atrium. Aorto-right atrial fistulas, abnormal connections between the aorta and the right atrium, are multi-factorial [2]. In this case report, we report a 37-year-old female patient with native aortic valve *Brucella* endocarditis and multiple vegetation on aortic and mitral valve, complicated by periannular aortic abscess perforation to surrounding tissue and cardiac chambers associated with acute heart failure, aorto-cavitary fistula, and left bundle branch block. She was scheduled for double valve replacement and pericardial patch repair of left valsalva sinus fistula to supra left atrial roof by pericardial patch use, and intra-cavitary closure of pulmonary fistula and intra-aortic repair of entrance site of fistula to left atrium which is described in this case report.

## Case report

**Figure 1:**
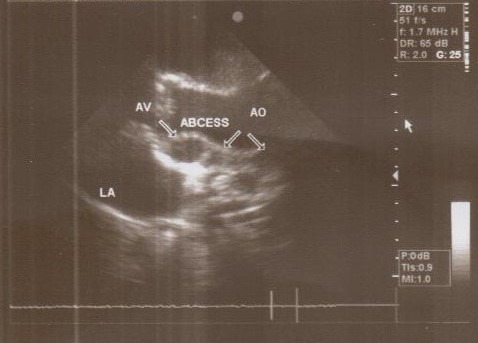
Abscess pocket over the left atrium

**Figure 2:**
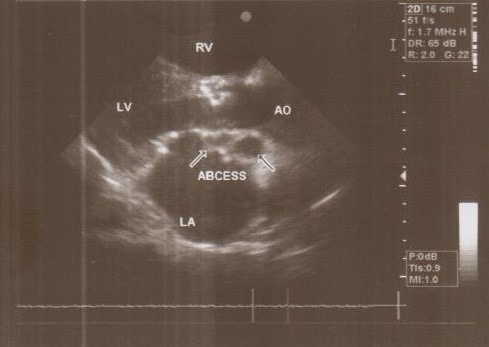
Multiple abscesses in periannular ring

**Figure 3:**
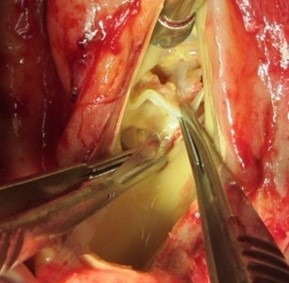
Destruction of three leaflets of aortic valve

**Figure 4:**
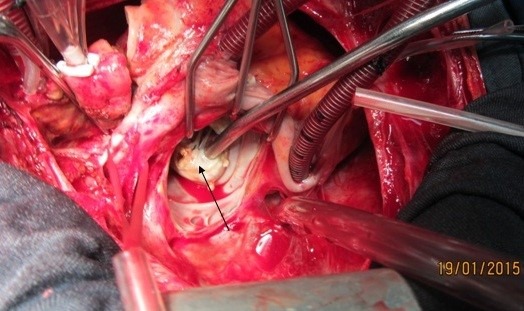
Vegetation on destructive anterior mitral leaflet (black arrow)

**Figure 5:**
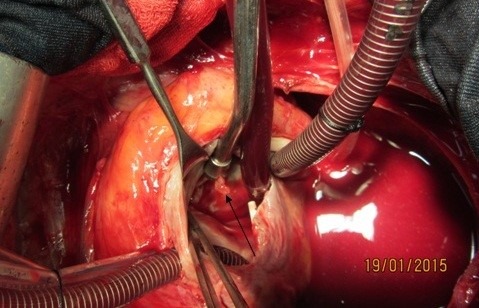
Vegetation in entrance of fistula tract to right atrium in tip of suction (black arrow)

**Figure 6:**
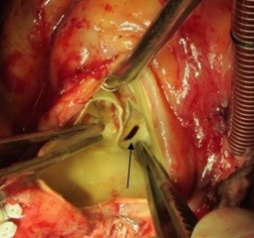
Right atrial fistula from non-coronary aortic sinus (black arrow)

**Figure 7:**
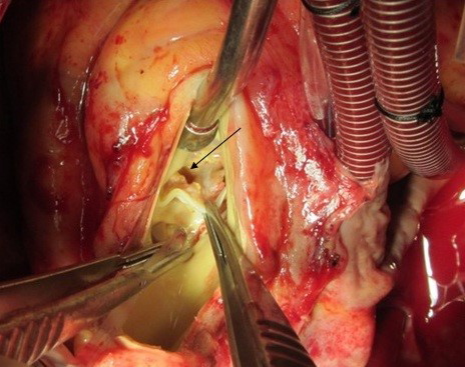
Fistula to pulmonary artery (black arrow)

**Figure 8:**
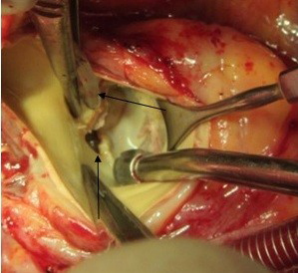
Left coronary defect imposing as a fistula to the left atrial roof (vertical arrow); cardioplegia catheter positioned at the left osmium (horizontal arrow)

A 37-year-old female patient was referred to our hospital following a full course of *Brucella* endocarditis in a general hospital for emergency surgery of *Brucella* endocarditis of aortic and mitral valve. The patient’s past medical history revealed living in endemic area of *Brucella* infection and a history of consumption of unpasteurized milk products. Her medical history was unremarkable except for sacroiliac arthritis. She had been admitted in a general hospital for assessing fever over the last month. After detecting a positive blood culture for *Brucella* without its bio-typing, she was treated by following triple combination of drugs consisting of oral rifampin 900 mg per day (qd), oral doxycycline 100 mg twice per day (bid) and gentamicin 80 mg intravenously three times per a day (tid) adjusted with blood levels of drugs and serum BUN and creatinine level. Upon admission, the patient was feverous, tachycardic, her blood pressure was low (80/20), and she had dyspnea. On neurologic examination, the patient was awake and oriented; her skin was cold and damp. The patient’s previous blood cultures at three different times showed a *Brucella* infection. White blood cell count: 12,000/mm^–3^ with 70% neutrophils, platelet count: 80,000/mm^–3^, hemoglobin: 9 g/dl, C-reactive protein: 60 mg/dl, erythrocyte sedimentation rate: 75 mm/h, blood urea nitrogen (BUN): 60 mg/dL, and creatine (Cr): 3.9 mg/dL. Urinalysis revealed no hematuria and 24-hour (diurnal) urinalysis (UA) revealed proteinuria. Serum agglutination tests were positive (titer>1:1,500), and enzyme-linked immunosorbent assay tests for anti-*Brucella* IgG and IgM antibodies were strongly positive (150 U/ml and 52 U/mL, respectively). A transthoracic echocardiogram (TTE) delineated destruction of mitral and aortic valves by multiple vegetation and multiple small and large aortic ring abscesses extended to surrounding tissue and perforated to right atrium, main pulmonary artery and formation a pocket over the left atrial roof (Figure 1 (Fig. 1), Figure 2 (Fig. 2)). The ejection fraction (EF) was 50% and pulmonary pressure was 60 mmHg. There was severe aortic, mitral and tricuspid valve regurgitation. The patient continued to use the previous anti*-Brucella* drugs orally while additional evaluations were performed. Due to the patient’s congestive heart failure (CHF) in addition to her multiple mobile aortic and mitral valve vegetation, it was decided that aortic and mitral valve replacement shall be performed immediately. The patient was scheduled for an emergency double valves procedure. However, the night before the surgery, she was intubated due to respiratory distress and was subsequently connected to mechanical ventilator. The patient suffered from severe pulmonary edema caused by CHF that required mechanical ventilation. After intubation, the patient became hypotensive and oliguric needing inotropic drugs use. The patient’s hemodynamic became stabilized and she was taken to the operating room. The intra-operative transesophageal echocardiogram (TEE) did not reveal any new findings. The patient was taken to operating room and a median sternotomy was performed and aortic and bi-cava cannulation was done. After opening the pericardium, it was found that the aortic root was severely attached to the surrounding tissue by inflammatory reaction caused by perforation of abscess in left coronary sinus to roof of left atrium as observed in TEE. The ascending aorta was cross-clamped, and after transverse transaction of the ascending aorta, cardioplegin was indirectly infused to coronaries ostium to induce cardiac arrest. After moderate hypothermic cardioplegic arrest, the umbilical tape was put around both the superior vena and inferior vena cavae and they were snared. The right atrium and left atrium were opened superior and inferior to the atrioventricular groove. Further, intra-operative inspection of right atrium showed small vegetations in crater of fistula entrance to right atrium in antero-medial region of tricuspid ring (Figure 3 (Fig. 3)). However, the tricuspid valve was not involved in infective endocarditis. Intra-aortic root inspection revealed a defect in non-aortic coronary sinus filled with necrotic materials and an abscess that perforated through the area above the tricuspid valve (Figure 4 (Fig. 4)). There was also a fistula between the left-coronary sinus, just near the left coronary ostium to the main pulmonary artery (Figure 5 (Fig. 5)). Further intra-operative perception of aortic root revealed a pocket filled by abscess through a defect in left coronary sinus just located over the roof of the left atrium (Figure 6 (Fig. 6)). In addition to the aforementioned fistulas, multiple vegetations were observed on both mitral and aortic valves that caused severe destruction of both valves causing grave regurgitation (Figure 7 (Fig. 7)). It apeared that mitral valve vegetations were caused by regurgitated aortic valve flow that impinged on aorto-mitral fibrous continuity and subsequently caused the penetration and destruction of the native mitral valve (Figure 8 (Fig. 8)). This infective tissue involved the valve. The abscess was completely debrided to restore and find underlying normal tissue. After debridement of the perforation’s site of the left coronary sinus and cleaning of the performed pocket over the left atrial roof, the aortic defect was repaired by fresh autologous pericardium patch that was used in the external side of the ascending aorta. The fistula tract to main pulmonary artery was closed from intra-pulmonary side of fistula by 4/0 proline sutures, as the closure of small fistula to right atrium. After closing of all three fistula and reconstructing the left sinus of valsalva and replacement of both valves, an oval-shaped fresh pericardial patch was utilized in a sino-tubular junction positioned just close to the superior vena cava, which helped in a tension-free approximation of aortotomy incision. Because the perforation of abscess along the conduction system caused bundle branch block and disturbances of other conduction branches, the atrial and ventricular epicardial pacing wires were used for sequential atro-ventricular pacing. The patient was admitted to the surgical intensive care unit for further control and treatment. Weaning from mechanical ventilation was complicated with tachypnea and grave respiratory distress. Extubation was delayed on the 9^th^ post-operative day after performing a tracheostomy for the better cleaning of respiratory tract secretion and facilitation of extubation. Transient renal and hepatic failure also complicated the postoperative course of surgery. The serum blood nitrogen and creatinine raised to 90 and 5.5 subsequently and was managed accordingly by three times of peritoneal dialysis. Her hepatic and kidney dysfunction recovered relatively in 15^th^ day of operation. A TEE in discharge time revealed a normal functioning of both bioprosthetic valves; however, a mild paravalvular leakage was observed in aortic position. Moreover, correction of all fistulas was successful and no residual flow signal in area of fistula repair was found. She was discharged home on the 25^th^ day after admission.

## Discussion

Brucellosis is a common infection in Middle East countries such as Iran and its transmission routes to humans include ingestion of infected milk products, or rarely direct contamination of mucous membranes, or inhalation of bacteria in respiratory system. Human brucellosis is a chronic multi-organ infection that may involve any body system and is characterized by systemic spreading to any other organ such as the pericardial space causing pericarditis, myocarditis, and endocarditis [3]. Involvement of the cardiac tissue is characterized by chronic and relapsing courses, and the incidence of mortality is less than 1% in non-cardiac brucellosis. However, 80% of the mortality of patients is caused by *Brucella* endocarditis [4]. Endocarditis has been estimated to occur in 11.5% of cases of body organ brucellosis. The in-hospital mortality rate of *Brucella* aortic endocarditis that is complicated by aortic annular abscess is estimated to be up to 30% that could increase to 50% if it was associated with intra-cardiac fistula [5]. The invasiveness of these bacteria has been also confirmed in *Brucella* prosthetic valve endocarditis. In our case, multiple risk factors were observed for increasing mortality including annular abscess rupture of valsalva sinus, presence of multiple fistula to cardiac chambers, large and mobile vegetation on both aortic and mitral valves, mitral, aortic, and tricuspid valves regurgitation, left to right shunt, and sepsis. All aforementioned complications are associated with high mortality and serious prognosis. However, experts recommend open surgery of complicated *Brucella* endocarditis (BE) within some days from diagnosis, but there is no consensus about the optimal time of surgery in BE [6], [7]. The present guidelines for the surgery of *Brucella* infective endocarditis are not straightforward as in non-*Brucella* endocarditis cardiac surgery has been recommended within a week from diagnosis [7]. The absence of sufficient evidence to define the optimal time of surgical intervention in BE is complicated by aortic annular abscess related to lack of available data about surgery of BE in the advanced stage. Most authors recommend a combination of prolonged medical therapy and early valve replacement combined with grave debridement of necrotic tissues, abscess’s spaces, vegetation for increased chance of successful treatment, and reducing rate of relapse of *Brucella* endocarditis. The combination of both medical and surgical treatments is obligatory, especially in those patients presenting with congestive heart failure related to grave aortic insufficiency caused by valve destruction [8]. Some authors also alleged that the risk of embolism by non-sessile, long and mobile vegetations could also need early surgical operation [9]. The indications for surgical intervention in *Brucella* endocarditis are similar with the existing guidelines of the treatment of infective endocarditis in general [10]. Valve replacement could be undertaken despite the existence of active disease, because the risk for prosthetic infection of the newly used device seems to be low [11], [12]. However, periprosthetic valve leakage is increased if the valve replacement is performed in the active phase of *Brucella* endocarditis. Other focal complications of valve replacement during active phase of disease related to periannular abscess formation, myocardial abscess formation, and valve dehiscence should be managed as infective endocarditis in general manner [13]. A wide range of other post-operative complications are observed in *Brucella* endocarditis surgery. Some of these complications are aortotomy site aneurysms, left ventricular failure by myocarditis, sepsis-induced disseminated intravascular coagulation, thrombo-embolic complications, stroke, and other organ infarctions [14]. One study demonstrated that surgical intervention undertaken within 72 h after documentation of *Brucella* endocarditis associated with valvular regurgitation and highly mobile and bulky vegetations leads to improved outcome [15]. Another study also reported a significant relation between early surgical intervention and survival [16]. Cerebral complications such as stroke if not associated with cerebral hemorrhage are not a contraindication for cardiac surgery. Neurological sequalae occur in approximately 30% of all patients with *Brucella* infective endocarditis. In the absence of intra-cerebral bleeding, surgical intervention should be undertaken with a low risk rate of 4% for neurological complications. Detection of an abnormal connection between valvular ring and cardiac chamber or the pericardium may or not lead to cardiogenic shock and should be considered as an inevitable indication for emergency cardiac surgery. Although the patient was intubated, hemodynamic instability of patient and her dependency to high dose inotrophic drugs caused us not to undertake CT scan to confirm suspected fistulas seen in TTE. Although TTE with high specificity and sensitivity detects fistula, only the CT scan could confirm the lesion and surgical planning. CT scan facility was not available in our center, so, to reduce further risk of hemodynamic deterioration, this patient was not transferred outside of hospital. Post-operative thrombo-emboli break-off from prosthetic valves vegetations or from native infected valve may cause septic infarcts of other organs, leading to relapse and prolonged fever [17]. However, the threat of thrombo-embolism is not higher than other types of endocarditis. Therefore, contrary to the conventional bacterial endocarditis, the size of vegetations frequently present in BE is bulky and large, as in our patient. Because no general consensus exists for the timing of antimicrobial agents administered in postoperative periods, clinical, periodical, serological, and microbiological exams may help the clinician to decide whether medical treatment should be continued or stopped. The normalization of IgG or IgM antibodies titers is sometime offered to be the final point for effective medical therapy [18], [19], [20], [21]. We think that prolonged postoperative triple or quadruple antibiotic treatment was indicated in our patient to protect the newly implanted prosthetic valves.

## Conclusion

The case of this 37-year-old female is unique, because it has some particularly interesting characteristics. First, triple valve infective endocarditis due to *Brucella* is exceedingly less frequent than other types of infective endocarditis. Another specific finding is that *Brucella* endocarditis was associated with multiple fistulas from three sinuses to three chambers. In this patient, the *Brucella* was so destructive that it caused periannular annular abscess and destruction of the aortic valve by vegetation, and the formation of multiple abscess that ruptured through the aortic ring to right atrial space, pulmonary artery, and to over roof of left atrium. Long-term antibiotic treatment of *Brucella* endocarditis before surgery was insufficient to eradicate the infection in our patient with aorto-cavitary fistula.

## Notes

### Competing interests

The authors declare that they have no competing interests.

## References

[1] Young EJ (1995). An overview of human brucellosis. Clin Infect Dis.

[2] Al-Harthi SS (1989). The morbidity and mortality pattern of Brucella endocarditis. Int J Cardiol.

[3] Peery TM, Belter LF (1960). Brucellosis and heart disease. II. Fatal brucellosis: A review of the literature and report of new cases. Am J Pathol.

[4] Alton GG, Jones LM, Angus RD, Verger JM (1988). Techniques for the Brucellosis Laboratory.

[5] Uddin MJ, Sanyal SC, Mustafa AS, Mokaddas EM, Salama AL, Cherian G, Mounjaeed M, Shuhaiber H (1998). The role of aggressive medical therapy along with early surgical intervention in the cure of Brucella endocarditis. Ann Thorac Cardiovasc Surg.

[6] Mert A, Kocak F, Ozaras R, Tabak F, Bilir M, Kucukuglu S, Ozturk R, Aktuglu Y (2002). The role of antibiotic treatment alone for the management of Brucella endocarditis in adults: A case report and literature review. Ann Thorac Cardiovasc Surg.

[7] Halim MA, Jeroudi MO, Mercer EN, Fawzy ME, Ziady G (1986). Infective endocarditis in King Faisal Specialist Hospital: A review of 35 consecutive adult patients. Ann Saudi Med.

[8] Cohen N, Golik A, Alon I, Zaidenstein R, Dishi V, Karpuch J, Zyssman I, Modai D (1997). Conservative treatment for Brucella endocarditis. Clin Cardiol.

[9] al-Kasab S, al-Fagih MR, al-Yousef S, Ali Khan MA, Ribeiro PA, Nazzal S, al-Zaibag M (1988). Brucella infective endocarditis. Successful combined medical and surgical therapy. J Thorac Cardiovasc Surg.

[10] Jacobs F, Abramowicz D, Vereerstraeten P, Le Clerc JL, Zech F, Thys JP (1990). Brucella endocarditis: The role of combined medical and surgical treatment. Rev Infect Dis.

[11] Colmenero JD, Reguera JM, Martos F, Sánchez-De-Mora D, Delgado M, Causse M, Martín-Farfán A, Juárez C (1996). Complications associated with Brucella melitensis infection: A study of 530 cases. Medicine (Baltimore).

[12] Alsoub H (2001). Brucella infective endocarditis: A report of four successfully treated patients. Clin Microbiol Infect.

[13] Cakalagaoglu C, Keser N, Alhan C (1999). Brucella-mediated prosthetic valve endocarditis with brachial artery mycotic aneurysm. J Heart Valve Dis.

[14] Fernandez-Guerrero ML, Martinell J, Aguado JM, Ponte MC, Fraile J, de Rabago G (1987). Prosthetic valve endocarditis caused by Brucella melitensis. A report of four cases successfully treated with tetracycline, streptomycin, and sulfamethoxazole and trimethoprim plus valve replacement. Arch Intern Med.

[15] Arslan H, Korkmaz ME, Kart H, Gül C (1998). Management of Brucella endocarditis of a prosthetic valve. J Infect.

[16] Reguera JM, Alarcon A, Miralles F, Pachon J, Juarez C, Colmenero JD (2003). Brucella endocarditis: Clinical, diagnostic, and therapeutic approach. Eur J Clin Microbiol Infect Dis.

[17] Lezaun R, Teruel J, Maitre MJ, Artaza M (1980). Brucella endocarditis on double valvular prosthesis. Postgrad Med J.

[18] Keles C, Bozbuga N, Sismanoglu M, Güler M, Erdoğan HB, Akinci E, Yakut C (2001). Surgical treatment of Brucella endocarditis. Ann Thorac Surg.

[19] O’Meara JB, Eykyn S, Jenkins BS, Braimbridge MV, Phillips I (1974). Brucella melitensis endocarditis: Successful treatment of an infected prosthetic mitral valve. Thorax.

[20] Chan R, Hardiman RP (1993). Endocarditis caused by Brucella melitensis. Med J Aust.

[21] Iglesias A, Nunez L (1981). Brucella melitensis endocarditis on a porcine heterograft bioprosthesis. Chest.

